# Supplementary material for: Are carbon emissions trading and green financial instruments synergistic? -Comprehensive quantitative research based on content analysis
Source: PLoS One. 2024 Mar 7;19(3):e0298601. doi: 10.1371/journal.pone.0298601 (PMC10919601; doi:10.1371/journal.pone.0298601)
Supplement: S1 Data — (ZIP) [file pone.0298601.s001.zip › Data/地级市Z_F工作报告.zip!/╒π╜¡/└÷╦«╩╨/└┤╫╘╣1⁄2╓┌║┼í╛┬φ┐╦╩2╛▌═°í┐_2014.docx]

各位代表：

现在，我代表市人民政府向大会作政府工作报告，请予审议，并请市政协委员和其他列席人员提出意见。

一、2013年工作回顾

2013年，面对复杂多变的国际国内形势，全市上下在省委、省政府和市委的坚强领导下，坚持以科学发展观为统领，稳增长、调结构、促改革、惠民生，较好地完成全年目标任务，全省排名的35项主要经济指标增速，有26项居全省前三。全市实现生产总值983.1亿元，增长9.2%；财政总收入124.2亿元，其中地方财政收入73.7亿元，分别增长10.3%和14.1%；城镇居民人均可支配收入29045元，名义增长10.4%；农村居民人均纯收入10024元，名义增长13.2%；节能减排预计完成省级下达目标。

（一）紧紧围绕稳中求进的总基调，积极应对，牢牢把握经济工作主动权

项目建设扎实推进。重点实施55个省“411”重大项目和220个市重点建设项目，全市完成固定资产投资570.4亿元，增长20.9%。云景和龙庆高速公路建成通车，实现县县通高速。金丽温铁路扩能改造、龙浦高速公路、浙北至福州特高压输变电、金丽温天然气管道、独流入海瓯江治理等一批项目加快实施。丽水机场、金台铁路、龙丽温（泰）高速公路等项目前期有力推进，衢宁铁路正式立项。

招商引资富有成效。引进大项目128个，其中5亿元以上大项目18个。全市实际利用内资237.9亿元、外资1.3亿美元，浙商回归到位资金70.2亿元，均超额完成年度计划。莲都、龙泉、遂昌、松阳等4家产业园被命名为首批省级山海协作产业园。

创新驱动不断见效。新增省级企业研究院和研发中心16家、国家重点支持高新技术企业39家、省级科技型中小企业92家，累计建成院士专家工作站15家，省生态经济研究院“落户”丽水。新增专利授权量4277件，其中发明专利84件。新增省级名牌和省级著名商标29件。荣获全国科技进步先进市称号。

企业帮扶积极实施。减免企业税费20.5亿元，兑付扶持资金13.5亿元。完成“小上规”111家，“个转企”4276家，股权交易挂牌企业40家。积极搭建银企合作平台，与国开行、省农行、浙江信保签订战略合作协议，金融机构本外币贷款余额1302.8亿元，增长16.5%。出台外贸稳增长政策，外贸进出口总额25.9亿美元，增长15.6%，出口超千万美元企业达到55家。

（二）紧紧围绕转型升级的主线，突出重点，加大重大产业平台建设

生态农业在彰显特色中稳步提升。推进农业“两区”建设，新建粮食生产功能区5.6万亩、现代农业综合区2个、主导产业示范区6个、特色农业精品园9个、生态精品林业基地38.6万亩。新增农村土地流转面积14万亩，新发展家庭农场1085家。新增绿色、有机和无公害认证农产品98个。

工业经济在加快转型中持续增长。制定促进工业转型升级5大类23项专项资金政策，推进生态产业集聚区和10个产业集群示范区建设，优化丽缙五金科技产业园市县共建体制。全市规模以上工业总产值、增加值和新产品产值分别增长12.9%、12.6%、51.2%，战略性新兴产业增加值增长14.1%，增速均居全省第一。工业技改投资113.6亿元，增长35.2%。

生态服务业在融合协调中加快发展。丽水现代商贸中心等4家服务业集聚区成为省级服务业集聚示范区，丽水无水港等8个项目被列入全省百个重点物流发展项目。电子商务、文化创意、信息、科技等新兴服务业方兴未艾。遂昌成为省首批电子商务示范县。全市服务业增加值和社会消费品零售总额均突破400亿元，分别增长8%和15.4%。

生态旅游和休闲养生（养老）产业在扩量提质中齐头并进。成功举办首个丽水生态文化旅游季，古堰画乡、中国青瓷小镇创建国家4A景区和松阳田园风情省级旅游度假区通过评审。全市实现旅游总收入266.3亿元，增长29.4%。重点推进18个养生（养老）基地项目，启动创建农家乐综合体26家。荣膺全国首个地级市“中国长寿之乡”。

（三）紧紧围绕全面深化改革的新要求，攻坚破难，致力山区科学发展综合改革

三大全国改革试点深入实施。坚持把重大政策问题突破作为基本目标，农村金融改革试点方面，基本构建农民住房财产权、林权、土地流转经营权“三权”抵押贷款体系，累计发放“三权”抵押贷款11.9万笔、114亿元，年末贷款余额44.9亿元，其中林权抵押贷款余额36.6亿元，占全省总量的61%。村镇银行和小额贷款公司实现县（市、区）全覆盖。低丘缓坡综合开发利用试点方面，累计完成土地征收5.2万亩、场地平整4万亩，通过国土部试点中期评估。扶贫改革试验区方面，确定新一轮扶贫重点村1569个，完成异地搬迁农民2.1万人，来料加工扶贫模式入选中国社会扶贫创新“八大样板”。

一系列热点、难点问题得到逐步解决。完善工程建设招投标管理办法，统一规范全市招投标市场。妥善处置企业资金链风险，集中化解银行不良资产7.5亿元。市区欧陆风情园建设、老机场地块开发、新机场选址、农贸市场改造提升、瓯江航道开发、天宁公租房建设等一系列社会关注度较高的问题，通过集中攻坚、创新方法得到逐步解决。

（四）紧紧围绕“秀山丽水、养生福地、长寿之乡”的区域定位，开拓进取，推进城乡统筹建设和管理

城市品位持续提升。深化“六城联创”，成功创建国家园林城市，国家卫生城市创建通过省级暗访，省级历史文化名城通过专家评审。突出“六个一”项目建设，8个县城基础设施、商业配套加快完善，特色优势进一步彰显。突出“三改一拆”，全市拆除违法建筑767.8万平方米，改造旧住宅、旧厂区、城中村988.4万平方米，省级下达三年任务一年完成。突出城市治堵，市区新增停车位8022个、公交车40辆、公共自行车3000辆。

垟

新农村建设纵深发展。开展“六边三化三美”行动和历史文化村落保护利用，新创市级美丽乡村示范村20个，完成村庄综合整治208个。龙泉上 入选十大中国最美小镇。莲都大港头、云和紧水滩入选全省十大最美乡镇。

生态文明建设成效显著。生态环境状况指数连续十年居全省首位、全国领先，丽水市和遂昌、云和同时列入全国生态文明建设试点。遂昌、庆元国家级生态县通过现场验收。

（五）紧紧围绕改善民生的根本目的，创新管理，财政支出七成以上用于民生事业

社会保障不断完善。全市财政民生支出144.5亿元，增长18.7%，占财政总支出的74%。新增养老、医疗、工伤、生育、失业保险19.2万人，基本医保实现城乡一体化。新建城乡社区养老服务照料中心304个，市儿童福利院、处州福利中心建成投入使用。新增城镇就业2.7万人，城镇登记失业率2.96%。新建保障性住房2858套。

社会事业全面发展。新创义务教育标准化学校57所，高考第一批上线2639人，增加476人，创历史新高。成功引进5家省级优质医院合作办医，市级公立医院综合改革启动实施，市中心医院综合病房大楼和6个县级医院迁建扩建项目基本竣工。成功举办国际摄影文化节、国际轮滑公开赛等重大文体活动。工青妇、人口计生、民族宗教、侨台外事、慈善等事业取得新进展。

社会环境和谐稳定。创新社会管理与建设，加强矛盾纠纷排查与化解，所有乡镇（街道）平安创建达标，安全生产事故总数、死亡人数和直接经济损失连续十年“三下降”，食品安全群众满意率连续五年全省第一，群众安全感满意率97.5%，有望夺取省平安银鼎。国防动员、人民防空、双拥优抚等工作取得新成绩。

（六）紧紧围绕市委大兴干事文化的总要求，改进作风，加强政府执行力建设

行政效能逐步提高。推进行政审批“四减少”改革，市本级审批事项精简为403项。组建投资项目审批代办中心，签约代办项目41个，总投资60多亿元。着眼于理顺关系、提高效率，下放莲都行政事权57项，划转编制人员237名。市县食品药品和工商、质监管理体制优化整合有序推进。“智慧政务”试点完成主平台搭建。开办全国首个常态化电视问政节目。

民主法制进一步加强。坚持重大事项向人大报告和政协通报制度，交由政府系统办理的208件人大代表建议和418件政协提案全部办结。开展依法行政示范单位创建活动，推进省行政调解试点。村级组织换届基本完成。坚决落实中央“八项规定”和省委“六个严禁”，加强廉政风险防控，“三公”经费支出下降14.3%。

各位代表，过去一年的成绩，来之不易，这是市委正确领导的结果，是全市人民团结拼搏的结果，是社会各界共同努力的结果。在此，我代表市人民政府，向全市各族人民和建设者，向人大代表、政协委员，向各民主党派、工商联、人民团体和社会各界人士，向驻丽部队官兵和省部属驻丽各单位，向关心和支持丽水发展的海内外朋友们，表示衷心的感谢！

我们也清醒地看到，政府工作还有不少差距：一是生产总值、工业产值、农民收入等指标与预期目标还有差距，特别是经济总量仍然偏小，提升区域综合竞争力任重道远。二是生态产业距离成为全省乃至全国示范还有差距，特别是生态工业仍处于启动阶段。三是破解发展新问题仍需付出更大努力，特别是各项改革试点，还需不断地取得阶段性的成果。四是政府工作抓“点、线、面”的意识、苦干实干的劲头仍需强化，“四风”问题在一些部门和地方依然存在，特别是项目的开工率仍有待提高、完成进度也有待进一步加快。对此，我们将高度重视，切实加以改进。

二、2014年主要目标和任务

2014年是全面深化改革的第一年，也是实施“十二五”规划的攻坚之年，改革发展任务重大而艰巨。世界经济仍将延续缓慢复苏态势。国内经济，一方面是十八届三中全会作出全面深化改革的决定，将进一步激发市场主体活力；另一方面，增长速度的换档期、结构调整的阵痛期、前期刺激政策的消化期“三期”叠加，稳中有忧，区域竞争更趋激烈。

综观国际国内经济形势，我们既要看到挑战和压力，坚持底线思维，更要看到优势和机遇，坚定发展的信心。我们坐拥全省乃至全国领先的好生态、好环境，我们有三大先行先试的“全国改革试点”，我们的“水陆空”立体交通网络在加速建设，我们的华侨优势、人文优势、后发优势和处于长三角、海西区结合部的区位优势在不断凸显。我们必将迎来属于丽水的“大时代”！对此，我们必须有充分的自信，迎难而上，乘势而上，实干而上，努力夺取新一轮发展的主动权。

2014年政府工作的指导思想是：认真贯彻党的十八大和十八届三中全会以及省委十三届四次全会、市委三届六次全会精神，坚持“绿水青山就是金山银山”的绿色生态发展理念，以全面深化改革为动力，以建设美丽幸福新丽水为目标，以“三区两城”创建为载体，加快构建“十个最”的体制机制，加快市域治理体系和治理能力现代化，加快“绿色崛起、科学跨越”，努力在生态文明建设上走在全国全省前列。

建议今年经济社会发展的主要预期目标为：地区生产总值增长9.5%；地方财政收入增长8%；城镇居民人均可支配收入名义增长10%以上，农村居民人均纯收入名义增长12%以上；固定资产投资增长20%；工业增加值增长11%以上；出口总额增长7.5%；社会消费品零售总额增长13%；城镇登记失业率控制在4%以内；节能减排指标均按省定目标要求安排。

根据上述指导思想和预期目标，做好今年的政府工作，必须认真贯彻市委关于全面深化改革的决定，把改革创新贯穿于经济社会发展的各个领域。重点做好十个坚持，落实十项举措，全力办好全市十方面大事和市区十方面实事：

（一）坚持全面深化改革，着力推进“五大改革”新突破

深化全国低丘缓坡开发利用试点。坚持保质量和保进度并重，完成试点区块90%的开发任务，加快配套基础设施建设，着力创造“地等项目、地等企业”的条件。深化土地供给机制改革，建立“能上能下、能进能出”的企业用地机制和供地负面清单，实行以“亩产论英雄”定地价、以约束性指标定奖惩，提高土地利用水平。

深化全国农村金融改革试点。以构建普惠型农村金融服务为目标，推进城乡一体化信用体系建设、产业链延伸贷款、小额贷款保证等工作，积极促进农村“三权”抵押贷款扩面增量，新增“三权”抵押贷款10亿元以上。完善银政企合作平台，鼓励企业扩大直接融资，扶持优质企业加快股改、挂牌和上市。推进全国保险服务民生示范区建设。年末金融机构本外币贷款余额突破1500亿元。

深化全国扶贫改革试验区建设。加强国家扶贫开发政策的研究与对接，创新搬迁扶贫、产业扶贫和社会扶贫体制机制。积极推进农村集体资产股份制改造，引导农村土地承包经营权、集体经济股权、农民住房财产权规范流转，探索建立农村产权流转交易平台。加大对景宁畲族自治县以及民族乡镇、民族村的扶持。

深化全国生态文明建设试点。编制生态文明建设规划，确保省级生态市通过验收，积极争创国家生态文明先行示范区。科学修编市县生态环境功能区规划，实行差别化区域开发管理政策，优化和完善市域功能布局，加大主体功能区建设。加快生态资源资产化改革，推进排污权有偿使用与交易，探索建立生态效益评估与碳减排交易机制。

深化行政审批制度改革。继续精简下放一批行政审批和民生服务事项，完善市、县、乡、村四级便民服务体系。推行审批权力清单制度，培育和规范中介服务业，加快构建市县联动、部门联动的重大投资项目审批大代办平台。推进“智慧政务”试点，完成联网审批系统开发，加快政务云平台覆盖扩面。

（二）坚持“五水共治”，着力展现“秀山丽水、养生福地、长寿之乡”新形象

突出推进重点流域、重点地区污水整治。健全市、县、乡三级全覆盖的“河长制”体系，制定实施河道“水岸同治”的综合治理模式，组织“万名干部到基层抓治水”行动，力争在全省率先消灭黑河、率先治好臭河、率先清理垃圾河。农村污水处理行政村覆盖率70%以上。强力推进合成革、化工、钢铁、电镀等重点行业治污减排，对经过整治仍不能达标的企业，坚决予以关停。加大市区和8个县城内河整治，新建城镇污水处理管网90公里。

联动推进防洪水、排涝水、保供水、抓节水。加快防洪排涝工程建设，完成独流入海瓯江干堤建设12公里、中小河流治理60公里，开工建设青田水利枢纽，除险加固山塘水库199座。加强江河源头、湿地资源体系和饮用水源保护，力争完成60%县级以上城市备用水源地建设。积极推进工业节水和生活节水。

统筹推进环境综合整治。深化生态公益林、景观林、防护林建设，重视水土流失防治与矿山生态治理，牢筑浙江生态屏障。加强耕地污染治理与修复，推进烟尘控制区建设。继续加强城市治堵，坚持公交优先，倡导绿色出行。继续推进“六边三化三美”和“三改一拆”，建立健全长效机制。创建无违建县1个。

（三）坚持项目化推进，着力增强区域发展新动力

力促项目管理和实施。坚持“抓项目就是抓发展、抓发展必须抓项目”的理念，强化项目化推进的基本方法，以更多的大项目、好项目推动经济持续发展。完善重大项目领导跟踪协调、责任部门全程服务和“四办”联合督办制度，提高项目供地、环评、规划等环节的服务效率。全市计划安排重点建设项目230个，年度计划投资200亿元。加紧谋划、包装、储备一批事关长远发展的大项目，突出抓好130个重大前期项目，总投资1000亿元。

力促重大基础设施项目。围绕建设畅通便捷之城，抓好龙浦高速公路建设和龙丽温（泰）、武松龙、遂江、庆寿等高速公路项目前期，推进330国道莲都段等8个国省道项目建设，加快构建“两纵两横三支”高速公路网和国省道网络。基本完成金丽温铁路扩能改造路基工程，推进衢宁、衢丽、金台、龙浦、温武等铁路项目前期，加快构建“两纵两横”铁路网。启动瓯江航运开发，加快丽水机场项目前期和招商。抓好金丽温天然气管道、浙北至福州特高压输变电等项目建设。

力促产业项目有效投入。切实提高项目供地率、开工率、竣工率、投产率、产出率，促进一批重大项目签约落地、一批重大项目开工建设、一批重大项目建成投产。全市工业投资突破200亿元，其中制造业投资165亿元，均增长20%。加大经营性土地出让和普通商品房开发投资，促进房地产市场健康有序发展。创新矿产资源综合开发投资机制，努力盘活矿产资源。

力促民间投资。加大破除民间投资隐性壁垒，进一步激发民资、侨资活力。探索建立社会化投资服务机制，努力突破民资“投资难”与中小企业“融资难”的两难问题。支持民营企业参与国有企业改革重组，发展混合所有制经济。鼓励民营企业建立现代企业制度，合理引导兼并重组、强强联合。完善“进村入企”、“千名干部联系千家企业”长效机制，加大困难企业帮扶。

（四）坚持转型升级，着力打造“3+3”生态产业新优势

把生态农业作为农民增收的主攻方向。深入实施生态精品农业“361工程”，推进农产品质量安全可追溯体系建设。新建粮食生产功能区6.1万亩、现代农业园区9个、生态精品林业基地35万亩，新培育生态精品农业示范企业和示范合作社50家、示范家庭农场300家。加快农产品精深加工园区规划建设。重视打造“丽水生态精品农产品”区域品牌，加大培育网上营销市场和农村电子商务，推动更多的农产品上市交易。

把生态工业作为构建生态产业体系的启动点。编制生态制造业发展导向和布局指南，细化主导产业与空间布局规划，明确鼓励类、允许类、限制类、禁止类行业和项目。发布项目负面清单，坚决摒弃污染难以治理的工业项目。根据发展生态工业的特别规定性和各地自然禀赋，组织创建一批生态工业试点，实施差别化政策扶持和资源要素倾斜保障机制。完善生态产业集聚区突出“姓工”的体制机制，重点推进核心区块以及东扩区块、丽缙五金科技产业园、丽景民族工业园建设，加快打造工业机器人、生物制药、装备制造业等特色基地，积极创建国家级开发区。整合提升10个省级开发区，实行以企业亩产为核心的综合评价体系，推动特色块状经济向现代生态产业集群转型发展。

把生态服务业作为推进城市化的重要动力。整合服务业发展引导政策和专项资金，大力发展各类生产性和生活性服务业，促进服务业与新型城市化的融合发展。加快丽水现代商贸中心、家居生活广场、国际汽车城等重大服务业项目建设，启动实施丽水生态农林产品物流中心、庆元香菇市场物流中心等一批现代物流项目。科学规划发展港航经济、空港经济。积极推进一批总部经济基地建设，扶持培育本地优势企业总部经济，大力引进企业总部、区域性总部和各类结算中心。

把生态旅游业作为第一战略支柱产业来培育。完善旅游发展政策，创新旅游管理体制，争创省级生态旅游综合改革试验区。推动更多的旅游景区、度假区提升“晋级”，加快瓯江莲都段生态景区、缙云仙都、遂昌金矿、青田石门洞创建国家5A级旅游景区步伐。注重整合提升瓯江和千峡湖沿线重点旅游项目，启动青田北山旅游小镇建设。大力发展乡村休闲旅游，新建农家乐综合体10家。加大旅游对外宣传推介，提升旅游服务水平。全市旅游总收入增长25%。

把生态休闲养生（养老）产业和文化特色产业作为生态经济可持续发展的有力支撑。深化“五养”行业标准的研究与制定，加快通济堰养生文化园等十大养生（养老）基地和100个养生乡村建设，积极推出一批各具特色的观光型、体验型、避暑型养生（养老）项目。加快十大文化产业园区集聚化、特色化建设，推进古堰画乡油画产业基地等一批重点文化产业项目。总结提升十大特色文化品牌，办好一批重大文体活动。

（五）坚持创新驱动，着力完善自主创新和科技进步新体系

健全创新体系。积极培育创新型企业，鼓励企业开展产学研合作，提升关键技术、共性技术研发水平。力争创建国家级企业技术中心“零突破”，新建省级企业研究院和研发中心10家以上，新增高新技术企业和科技型中小企业100家以上。

建设创新平台。推进生态科技城规划和建设，扶持创建更多的省级创新服务平台，加快创建中药材、食用菌、特色机械装备等国家级创新服务平台。争取建成合成革、汽车空调等一批省级产品质检中心。积极促进工业化和信息化“两化”融合，加快绿谷信息产业园、海创园建设。

推进“四换三名”。积极推进腾笼换鸟，加快淘汰落后产能，组织实施100个以上战略性新兴产业投资项目，战略性新兴产业和高新技术产业增加值分别增长12%和10%。积极推进机器换人，鼓励企业加大技改投入，重点实施100个以上工业技改项目。积极推进空间换地，开展城镇低效用地再开发试点，加大标准厂房建设，新开发地下空间40万平方米。积极推进电商换市，扶持做大做特一批电子商务平台，实现电子商务销售额75亿元。坚持质量强市、标准强市、品牌强市，积极实施名企、名品、名家“三名工程”，推动龙头骨干企业和制造业50强向“五型企业”发展。新增专利授权量4000件，新增省级名牌和著名商标20件，参与制定修订国家和省级标准、行业标准4项。

（六）坚持在更大的范围集聚生产要素，着力构建开放型经济新体制

提升招商引资实效。完善招商引资指导性政策，强化产业链招商、侨资组团信托招商、生态资源优势招商、民族政策招商、装备制造业和战略新兴产业招商，加强与项目相关联的政府性机构、行业协会的联系合作，着力引进一批能够调结构、促转型、增后劲的生态经济大项目、好项目。争取引进国际国内500强、央企国企、浙商侨商回归和20亿元以上项目有新突破。全市实际利用市外内资220亿元，浙商回归到位资金75亿元。

深化区域合作和山海协作。优化山海协作共建机制体制，推动4个省级山海协作产业园建设和试点扩面。力争签约山海协作项目100个，到位资金120亿元。全面深化与长三角、珠三角以及海西区的交流合作，积极对接上海自贸区和全省“四大国家战略”，加强对台经济文化交流。

推动外资、外贸和外经、外包增量提质。创新外资利用方式，加强外资项目的包装和推介，大力引进和承接优质外资项目。全市实际利用外资1.5亿美元。创新外贸发展方式，加强出口基地和品牌培育，支持企业引进海外先进装备、技术和管理经验。鼓励优势企业扩大对外投资，支持更多企业参与服务外包行业。加强对外友好交流，以外事侨务促进外经贸发展。

加强招才引智和人才队伍建设。实施绿谷精英和创业创新团队引领计划，加大引进国家和省“千人计划”、科技领军人才。推进国家大院名校丽水产业科技服务中心、院士工作站和海外高层次人才创业创新基地等平台建设，鼓励和扶持高层次人才创业创新。健全人才培养和选拔机制，加强突出贡献专家以及高技能人才队伍建设，研究发挥“本土人才”作用的政策。

（七）坚持以更高的标准建设中心城市，着力打造宜居宜业宜游新城市

高起点规划城市发展。按照“一江双城”的总体框架，深化城市特色研究，基本完成新一轮城市总体规划修编，促进城市空间合理布局、公共设施集约建设、资源要素优化配置。深化各专项规划设计，编制历史文化名城保护规划，谋划打造一批富有地域特色和文化内涵的城市精品亮点。

加快完善城市功能。优化城市路网体系，推进花园路综合整治和富岭高速出入口改造提升，启动高速丽水北互通建设，新建续建联城路、教工路、城北路西段、大猷街西段、水东路二期、市场2号路等道路项目。推进旧机场、联城等区块道路框架和配套道路建设，加快富岭区块开发，加大北城道路综合整治。加强环南明湖旅游资源整合与开发，加快实施处州府城、欧陆风情园二期等一批项目，增强城市旅游休闲功能。

强化市区联动共管机制。建立市区城市管理快捷高效指挥协调机制，优化整合城市公共管理资源，提升网格化、精细化管理水平。完善城市管理各类长效机制，实施环境卫生责任区制度，增进市民文明卫生意识。牢牢把握创建关键环节，确保创成国家卫生城市，国家环保模范城市基本达标。

（八）坚持统筹发展，着力开创山区新型城市化、城乡一体化发展新局面

积极创建“最美县城”。以“六个一”项目建设为主载体，推进8个县城特色化、品质化发展。突出龙泉剑瓷风韵，加快建设浙闽赣三省边际区域重要节点城市。突出青田欧陆风情、石雕主题，加快建设最美侨乡。突出“老云和、新童话、真山水”，打造童话休闲旅游城。突出庆元廊桥、香菇特色，加快接轨海西区。突出缙云黄帝文化底蕴，积极接轨浙中城市群。突出遂昌黄金、汤显祖文化，加快建设长三角休闲旅游城。突出松阳田园风光，打造新型田园生态城市。突出景宁畲族风情，建设“神奇畲乡、休闲胜地”。

培育发展中心集镇。完善小城市和中心镇发展机制，加强以“四网二厂一园”为主的基础设施建设，加大培育22个省市级中心镇，推进壶镇、温溪、碧湖等3个小城市试点。深化强镇扩权改革，创新行政管理、财政金融、公共服务等体制机制，促进中心镇人口集中、产业集聚、功能集成。

深化建设美丽乡村。统筹推进“百村示范、千村整治”和历史文化村落保护利用，加大连片连线美丽乡村精品区域培育力度。抓好50个省级农房改造示范村建设，新建20个市级美丽乡村示范村，新打造10条美丽乡村风景线。促进城市基础设施和公共服务向农村延伸，加强农村联网公路建设，实现村村电气化。

（九）坚持转换理念，着力形成社会事业优质化发展新模式

创新社会事业投资机制。按照“保好基本、放开非基本”的原则，鼓励和引导民资办医、办学、办文化，完善政府购买服务制度，大力推进社会事业综合改革。深化与“大院名校”合作共建的体制机制，大力引进和拓展优质教育、卫生资源。积极扶持一批民办医院、民办博物馆落地建设。

创新社会事业建设机制。坚持适度超前的原则，完善城乡社会事业规划，增强与各领域规划的协调衔接。建立基本公共服务项目配套要素优先保障机制，积极推行“交钥匙工程”。推进丽水五中、市人民医院东城院区等项目，基本完成市第二人民医院迁建，实现市博物馆、体育馆开馆。

创新社会事业管理机制。深化国家和省各项教育改革试点，创新教育管理和教师队伍建设，切实重视高中教育的改革与发展，努力提升教育质量。支持地方高校和职业教育发展，增强服务地方发展能力。扎实推进公立医院改革，深化基层综合医改。加大农村教师、医师定向培养力度，完善更加体现行业特点的激励机制。健全城乡公共文化服务体系，深入实施文化惠民工程，广泛开展全民健身活动。坚持人口计生目标管理，实施“单独二孩”政策。

（十）坚持以人为本，着力构建和谐社会新格局

多渠道增加居民收入。深入开展农民增收“十大主题”活动。全面落实促就业各项政策，实现城镇新增就业1.4万人。逐步提高最低工资标准，有序推进企业开展工资集体协商，建立企业职工工资正常增长机制。健全完善社会救助体系，逐步提高城乡低保和农村五保供养补贴标准。

广覆盖完善社保体系。健全社保政策，基本构建“五保合一”大社保体系，稳步推进扩面提标。新开工建设保障性住房2500套。大力发展妇女、儿童、老龄、残疾人和慈善事业。鼓励和扶持民办养老、医养结合新模式，启动9个县（市、区）示范性综合养老院建设，新建城乡社区养老服务照料中心524个。开工建设市残疾人托养中心、怡福家园养老院。

深层次推进社会治理。培育和践行社会主义核心价值观，推进公民道德和社会诚信体系建设。深化网格化管理、组团式服务，健全外来人口管理服务体系，推进社会组织健康有序发展，创新信访工作机制，积极回应群众合理诉求，倾力化解积累性问题。完善社区管理体制，稳步推进“撤村建居”。加强安全生产和食品药品安全监管，健全防灾减灾救灾体制。着力化解企业债务危机和银行不良贷款。完善立体化社会治安防控体系，严厉打击违法犯罪活动。支持驻丽部队和国防后备力量建设，不断推进军民深度融合发展。

各位代表，民生是为政之要，今年我们将继续围绕事关人民群众切身利益的问题，坚持尽力而为、量力而行，努力办好全市十方面大事和市区十方面实事。全市十方面大事为治水工程、美丽县城、美丽乡村、生态农业、生态工业、生态旅游、交通网络、生态环保、要素保障、城乡安居。市区十方面实事为内河治理、小区改造、教育保障、养老保障、医疗保障、就业增收、扶弱济困、城市畅通、文化惠民、食品安全。

三、切实加强政府自身建设

以党的群众路线教育实践活动为契机，大兴干事文化，着力打造为民、务实、清廉的服务型政府。

（一）扎实开展党的群众路线教育实践活动。按照“照镜子、正衣冠、洗洗澡、治治病”的总要求，全面改进学风、会风、文风和工作作风。把学习教育、提高思想认识贯穿始终，树立正确的权力观、利益观，切实增强群众观点。把整改落实、解决实际问题贯穿始终，反“四风”，破“两局”。把建章立制、标本兼治贯穿始终，深化人事制度改革，健全改进作风常态化制度，努力培养和造就一支“小狮子型”的公务员队伍。

（二）大兴解放思想之风。把实事求是作为解放思想的内容和标准，努力做到“三问三思”、“三破三立”。把群众路线作为解放思想的根本路线，紧贴群众，问政于民、问需于民、问计于民。把全面建立“丽水自信”作为现阶段解放思想的重要内容，凡是有利于加快转型、推动开放、促进发展的改革目标、举措、制度、机制，都要敢想敢提敢争取，力争先行先试，创建更多的国家和省级改革试点。

（三）大兴真抓实干之风。强化“绿水青山就是金山银山”的定力、能力和执行力，树立“功成不必在我”的理念，勇于改革、勇于担当，努力干出气势。发扬踏石留印、抓铁有痕的工作作风，始终紧盯目标干，经常围着项目转，突出问题导向，解决棘手问题，努力干出新风。进一步理顺抓落实的机制体制，使每个单位、每位干部都有干的任务、都有干的责任，做到千斤重担合力挑、人人身上有指标，努力干出合力。坚持实干论英雄，深化考核激励机制创新，突出生态指数、发展指数、幸福指数，强化政府目标管理和绩效考评，努力干出导向。

（四）大兴为民服务之风。坚持“群众想什么，我们就干什么”的理念，把满足群众的需求、解决群众反映的问题、让群众满意作为努力方向，立说立行、见诸行动，让群众看到实实在在的成效和变化。深化政务公开和信息公开，加强行政执法监督和行政复议，切实保障公民合法权益。稳步推进新一轮机构改革，优化机构设置，理顺权责关系，提升服务效能。

（五）大兴清正廉洁之风。自觉接受人大、政协和社会各界监督，主动听取民主党派、工商联、无党派人士和人民团体意见。坚持民主集中制，健全行政决策科学化、民主化制度。深化惩治和预防腐败体系建设，坚持查处大要案和查处发生在群众身边的腐败问题并举，始终保持惩治腐败的高压态势。严格执行《党政机关厉行节约反对浪费条例》，“三公经费”预算支出削减30%。

各位代表，今年我们仍将面临许多困难，但更加充满机遇和希望。让我们在省委、省政府和市委的正确领导下，以“秀山丽水自图强”的更大信心和决心，振奋精神，改革创新，扎实苦干，坚定不移走“绿水青山就是金山银山”的绿色生态发展之路，为建设美丽幸福新丽水而努力奋斗！
